# A deep learning-based automated diagnostic system for classifying mammographic lesions

**DOI:** 10.1097/MD.0000000000020977

**Published:** 2020-07-02

**Authors:** Takeshi Yamaguchi, Kenichi Inoue, Hiroko Tsunoda, Takayoshi Uematsu, Norimitsu Shinohara, Hirofumi Mukai

**Affiliations:** a Division of Medical Oncology, Japanese Red Cross Musashino Hospital; b Breast Cancer Center, Shonan Memorial Hospital, Kanagawa; c Department of Radiology, St. Luke's International Hospital, Tokyo; d Division of Breast Imaging and Breast Interventional Radiology, Shizuoka Cancer Center Hospital, Shizuoka; e Department of Radiological Technology, Faculty of Health Sciences, Gifu University of Medical Science, Gifu; f Division of Breast and Medical Oncology, National Cancer Center Hospital East, Chiba, Japan.

**Keywords:** artificial intelligence, breast cancer, convolutional neural network, deep learning, mammography, screening

## Abstract

**Background:**

Screening mammography has led to reduced breast cancer-specific mortality and is recommended worldwide. However, the resultant doctors’ workload of reading mammographic scans needs to be addressed. Although computer-aided detection (CAD) systems have been developed to support readers, the findings are conflicting regarding whether traditional CAD systems improve reading performance. Rapid progress in the artificial intelligence (AI) field has led to the advent of newer CAD systems using deep learning-based algorithms which have the potential to reach human performance levels. Those systems, however, have been developed using mammography images mainly from women in western countries. Because Asian women characteristically have higher-density breasts, it is uncertain whether those AI systems can apply to Japanese women. In this study, we will construct a deep learning-based CAD system trained using mammography images from a large number of Japanese women with high quality reading.

**Methods:**

We will collect digital mammography images taken for screening or diagnostic purposes at multiple institutions in Japan. A total of 15,000 images, consisting of 5000 images with breast cancer and 10,000 images with benign lesions, will be collected. At least 1000 images of normal breasts will also be collected for use as reference data. With these data, we will construct a deep learning-based AI system to detect breast cancer on mammograms. The primary endpoint will be the sensitivity and specificity of the AI system with the test image set.

**Discussion:**

When the ability of AI reading is shown to be on a par with that of human reading, images of normal breasts or benign lesions that do not have to be read by a human can be selected by AI beforehand. Our AI might work well in Asian women who have similar breast density, size, and shape to those of Japanese women.

**Trial registration:**

UMIN, trial number UMIN000039009. Registered 26 December 2019, https://www.umin.ac.jp/ctr/

## 1 Introduction

Several randomized controlled trials have demonstrated reduced breast cancer-specific mortality due to mammography screening programs.^[1–3]^ The Japanese guidelines for breast cancer screening recommend mammography every 2 years for women aged 40 years and older.^[4]^ However the interpretative performance of diagnostic mammography is influenced by readers’ experience and working time engaged in breast imaging.^[5,6]^ The Japan Central Organization on Quality Assurance of Breast Cancer Screening has a program to evaluate mammography readers’ ability to maintain the quality of screening. The organization rates readers on a scale of A to D according to the sensitivity and specificity of mammography reading tests. Readers with rank A or B are considered to have above-average skills and are certified by the organization.

The breast cancer screening rate in Japan was 36.9% in 2016 (from Comprehensive Survey of Living Conditions), lower than that in other developed countries. Various efforts are being made to increase the rate. Increased numbers of women screened and the use of double reading in mammography screening programs, however, creates a high workload for readers and increases economic costs. Moreover, up to 25% of mammographically-visible cancers are still not detected at screening.^[7–10]^ Thus, alternative strategies are needed to reduce readers’ burden and detect breast cancer efficiently.

Computer-aided detection (CAD) systems have been developed to help doctors read mammographic images. However, the clinical utility of traditional CAD systems is not yet determined. While some studies have reported improved readers’ performance,^[11–14]^ others have shown increased false-positive results and unnecessary biopsies due to a low specificity.^[15–17]^

The field of artificial intelligence (AI) is rapidly evolving and novel CAD systems using deep learning convolutional neural networks have been developed. Several deep learning-based CAD systems for the analysis of mammograms have been developed, some of which have already shown very promising results.^[18–20]^ AI systems that have comparable reading ability to humans help readers improve the cancer detection rate as well as reducing the reading workload by pre-selecting suspicious lesions in mammographic images. However, the image analysis algorithms have been created and studied mainly in western countries. Because Asian women characteristically have higher-density breasts than women from other ethnic groups,^[21–23]^ it is uncertain whether AI systems created based on data from western countries can apply to Japanese women. Therefore, a deep learning-based automated diagnostic system for mammograms has to be developed with data from Japanese women.

In this study, we aim to construct a deep learning-based CAD system trained using a large number of mammograms from Japanese women. A large number of learning images with high-quality readings is crucial to create a sophisticated system. Thus, we will use the mammographic images read by readers ranked grade A according to the Japan Central Organization on Quality Assurance of Breast Cancer Screening.

This trial was approved by the institutional review board of National Cancer Center Hospital East.

## 2 Methods

### 2.1 Objectives

The aim of this study is to construct a deep learning-based AI system to detect breast cancer on mammograms with high specificity, and to evaluate the performance of the AI system.

### 2.2 Study setting

This is a multicenter retrospective study. We will use digital mammography (DM) images taken for screening or diagnostic purposes in participating institutions.

### 2.3 Endpoints

The primary endpoint is the sensitivity and specificity of the AI system to detect breast cancer with the test image set. Sensitivity is calculated as the number of images in which the AI system correctly diagnoses cancer among all images with biopsy-proven cancer. Specificity is calculated as the number of images in which the AI system correctly diagnoses normal or benign lesions among all images without cancer.

### 2.4 Eligibility criteria

#### 2.4.1 Inclusion criteria

DM images fulfilling all the following criteria will be collected.

1. Taken after 2010
2. Images meeting either one of the following criteria
  - Visible breast cancer or benign lesions on images.
  - Normal breast.
3. If cancer or benign lesions are visible on images, their outlines can be traced manually.
4. Images from patients aged 20 or older.
5. Available mediolateral oblique view with or without cranial-caudal view.
6. No visible axillary lymph node metastasis from breast cancer.
7. Images from patients with no previous history of chemotherapy, endocrine therapy or radiotherapy.
8. Images from patients who have not received any previous surgical breast procedure including partial resection, breast reconstruction, incisional biopsy, vacuum-assisted biopsy, and mammoplasty
9. Read by readers ranked A according to the Japan Central Organization on Quality Assurance of Breast Cancer Screening.
10. Benign lesions, breast cancer, and normal breast on images are confirmed by the following criteria.
  <Benign lesions>
  Meeting one of the following criteria
  - Confirmed by histopathology.
  - Without malignancy development over at least 2 years of follow-up.
  - Findings clearly indicating a simple cyst by mammography and other imaging modalities.<Breast cancer>
  - Confirmed by histopathology.
  <Normal breast>
  Meeting either one of the following criteria
  - In addition to the findings of mammography, ultrasonography and MRI do not detect any lesions.
  - Without malignancy development over at least 2 years of follow-up when no other imaging modalities except mammography are performed.

#### 2.4.2 Exclusion criteria

DM images fulfilling any of the following criteria will not be collected.

1. Tomosynthesis and synthetic 2D mammographic images
2. Spot compression views
3. Poor image quality
4. Inappropriate images as judged by the local investigators

### 2.5 Construction of an AI algorithm

The machine learning community has been applying deep learning in various medical imaging fields, including mammography images. Based on this work, we have developed a convolutional neural network. The neural network includes pairs of the convolutional layer and the pooling layer with the fully convolutional layers. The classification output is calculated by the softmax function.

From mammography images, corresponding masking images of breast lesions will be created manually, indicating the area of the lesions. Mammography images will then be cropped with rectangular patches so that the lesions are included in these patches, and patches will be classified as benign or malignant. Patches in which neither benign nor malignant tumors are included will also be cropped from the mammography images.

These patches will be randomly divided into the training dataset, the validation dataset, or the test dataset. The neural network will be fine-tuned to classify whether these patches contain either a benign or a malignant tumor, or neither of them.

### 2.6 Statistical analysis

The Breast Cancer Surveillance Consortium reported readers’ interpretive performance with sensitivity and specificity of around 85% and 90% respectively.^[24]^ The sensitivity of mammography was 77% in the J-SATRT trial conducted in Japan.^[25]^ Thus, we set the target sensitivity and specificity at 80% or more with the AI system. By referring to the design of previous studies.^[26]^ 15,000 DM images will be collected in this study. They will consist of 5000 images with breast cancer and 10,000 images with benign lesions. At least 1000 images of normal breasts will also be collected for use as reference data.

We will categorize images with breast cancer according to the main findings (mass, focal asymmetric density, calcification, architectural distortion). At least 750 images will be collected in each category.

There will be a limit on the number of accumulated images with each benign lesion. Simple cyst 1500; fibroadenoma or benign phyllodes tumor 1500; intraductal (intracystic) papilloma 1500; adenoma 100; adenomyoepithelioma 100; sclerosing adenosis 1000; mastopathy 1000; breast calcifications 4000; other benign disease 300 images.

## 3 Discussion

AI with deep learning needs good training data to work properly. Thus, we put a premium on the quality of training image data in the development of our AI. Experienced and qualified readers will interpret mammograms and define the outline of the malignant or benign lesions. To delineate the outline precisely, surgical pathology reports or the results of other imaging modalities will be taken into account whenever possible. The diagnosis will be confirmed by histopathology. Benign diseases without available pathological results will be clinically diagnosed after at least 2 years of follow-up. To avoid biased collection toward a particular lesion, a target number of images has been set according to each radiographic finding in breast cancer or in each benign disease.

Mammographic images will be obtained from multiple participating centers. The DM images will be acquired with devices from various vendors. The images used in this study will be taken from women aged 20 or older (not confined to the recommended screening age), and originating not only from screening but also from clinical practice. Larger and more advanced breast cancers are expected from clinical practice, with different characteristics from screen-detected cancers. These various kinds of data will increase the generalizability of our AI algorithms. Alongside images, we will collect clinicopathological features of each case, including pathological diagnosis, tumor diameter, hormone receptor status, HER2 status, breast density, and vendors. This will enable us to perform detailed analysis of which patient population is more suitable for AI reading.

Our study has a limitation. The dataset will come only from Japanese women. Thus, our AI system might not be applicable to Caucasian patients. However, it will probably work well in Asian women who have similar breast density, size, and shape to Japanese women.

Japan has a double reading system in screening mammography. When the ability of AI reading is shown to be on a par with that of human reading, images of normal breasts or benign lesions that do not have to be read by a human can be selected by AI beforehand (the first reader role). This AI preselection might obviate the need for double reading. However, future studies will be required to clarify how much the workload of readers is reduced or which threshold of cancer probability for alerting humans is optimal.

## Author contributions

**Acquisition of data and data analysis and interpretation:** Takeshi Yamaguchi, Kenichi Inoue, Hiroko Tsunoda, Takayoshi Uematsu, Norimitsu Shinohara, Hirofumi Mukai.

**Critical revision of the manuscript:** Kenichi Inoue, Hiroko Tsunoda, Takayoshi Uematsu, Norimitsu Shinohara, Hirofumi Mukai.

**Study concept and design:** Takeshi Yamaguchi, Kenichi Inoue , Hiroko Tsunoda, Takayoshi Uematsu, Norimitsu Shinohara, Hirofumi Mukai.

**Writing the manuscript:** Takeshi Yamaguchi.

All authors read and approved the final manuscript.
